# Identification of β**-**carboline and canthinone alkaloids as anti-inflammatory agents but with different inhibitory profile on the expression of iNOS and COX-2 in lipopolysaccharide-activated RAW 264.7 macrophages

**DOI:** 10.1007/s11418-018-1251-5

**Published:** 2018-10-15

**Authors:** Pan Liu, Huixiang Li, Ruiling Luan, Guiyan Huang, Yanan Liu, Mengdi Wang, Qiuli Chao, Liying Wang, Danna Li, Huaying Fan, Daquan Chen, Linyu Li, Keiichi Matsuzaki, Wei Li, Kazuo Koike, Feng Zhao

**Affiliations:** 1 Key Laboratory of Molecular Pharmacology and Drug Evaluation (Yantai University), Ministry of Education, Collaborative Innovation Center of Advanced Drug Delivery System and Biotech Drugs in Universities of Shandong, School of Pharmacy, Yantai University, Yantai, Shandong 264005 People’s Republic of China; 2 Faculty of Pharmaceutical Sciences, Toho University, Funabashi, Chiba 274-8510 Japan; 3 Pharmacy Dispensing Center, The Affiliated Yantai Yuhuangding Hospital of Qingdao University, Yantai, Shandong 264000 People’s Republic of China; 4 School of Pharmacy, Nihon University, Funabashi, Chiba 274-8555 Japan

**Keywords:** β-Carboline alkaloid, Canthinone alkaloid, Anti-inflammatory activity, NO, PGE_2_, iNOS, COX-2

## Abstract

A compound library, which consists of 75 natural β-carboline-type or canthinone-type alkaloids from Simaroubaceae plants and their chemical synthetic analogues, was screened for the anti-inflammatory activity by inhibition of the overproduction of inflammatory mediator nitric oxide (NO) in lipopolysaccharide (LPS)-activated RAW 264.7 macrophage cells. Six compounds, namely, benzalharman (**23**), kumujian (**27**), 1-ethyl-1,2,3,4-tetrahydro-β-carboline-3-carboxylic acid (**37**), 1-acetophenone-1,2,3,4-tetrahydro-β-carboline-3-carboxylic acid (**42**), cathin-6-one (**46**), and 9-methoxy-cathin-6-one (**57**), exhibited significant inhibitory activity on the overproduction of NO with good dose dependency. Further investigation demonstrated that all of the six compounds down-regulated the high expression of inducible nitric oxide synthase (iNOS) protein. Among them, two canthinone-type alkaloids (**46** and **57**) potently down-regulated cyclooxygenase-2 (COX-2) protein expression in a dose-dependent manner and also inhibited the overproduction of inflammatory mediator prostaglandin E_2_ (PGE_2_). However, the β-carboline-type alkaloids (**23**, **27**, **37**, and **42**) exhibited no obvious inhibition on the overproduction of PGE_2_ and the expression of COX-2 protein. The results suggested that β-carboline-type alkaloids and canthinone-type alkaloids may exert an anti-inflammatory effect through different mechanism.

**Electronic supplementary material:**

The online version of this article (10.1007/s11418-018-1251-5) contains supplementary material, which is available to authorized users.

## Introduction

Nitric oxide (NO) is a biological messenger molecule and neurotransmitter, which is synthesized by NO synthase (NOS) in multiple cells. Three types of NOS, namely neuronal NOS (nNOS), endothelial NOS (eNOS), and inducible NOS (iNOS), produce NO through the catalytic reaction of l-arginine and dioxygen. Different from nNOS and eNOS, iNOS is expressed only after being induced by extracellular stimuli such as stimulation with lipopolysaccharide (LPS) [1]. In addition, prostaglandin E_2_ (PGE_2_) is the main metabolite of arachidonic acid epoxy synthase, which is important for cell growth or regulation and can cause some symptoms of inflammation, such as swelling, redness, and heat [2]. In mammal cells, two types of cyclooxygenase (COX-1 and COX-2) have been identified [3]. COX-2, the key enzyme in the process of PGE_2_ synthesis, is highly expressed during the process of inflammatory reaction induced by LPS [4]. Many studies have reported that the high expression of iNOS and COX-2 promotes the overproduction of NO and PGE_2_ in activated macrophages, respectively. Excessive production of such inflammatory mediators and high expression of inflammatory proteins can result in chronic inflammatory diseases [5–7]. Chronic inflammation has also been linked with various diseases, such as systemic lupus erythematosus, rheumatoid arthritis, hepatitis, cancer, and so on [8–11].

β-Carboline-type alkaloids and canthinone-type alkaloids represent the major bioactive constituents of medicinal plants belonging to the Simaroubaceae family, and a large range of bioactivities have been reported due to the diversity of chemical structure [12–16]. For example, β-carboline-1-propionic acid and 1-hydroxy-canthin-6-one from *Ailanthus altissima* have been reported to possess potent inhibitory activity against cyclic adenosine monophosphate phosphodiesterase [17]. 4,5-Dimethoxy-10-hydroxy-canthin-6-one, canthin-6-one, 8-hydroxy-canthin-6-one, 4,5-dimethoxy-canthin-6-one, and 5-hydroxy-4-methoxy-canthin-6-one from *Picrasma quassioides* have been reported as having cytotoxic activity against human nasopharyngeal carcinoma (CNE2) cells [18].

During our continuing search for bioactive compounds from natural resources, we have established a compound library, which consists of alkaloids from Simaroubaceae plants, such as *Picrasma quassioides*, *Picrasma javanica*, *Ailanthus altissima*, *Simarouba amara*, *Eurycoma longifolia*, *Simaba cuspidata*, and *Quassia amara*, as well as their chemical synthetic analogues. The chemical structures are shown in the Supplementary data, which included 38 β-carboline alkaloids (**7**–**45**), 31 canthinone alkaloids (**1**, **3**–**6**, and **46**–**70**), and 6 dimeric alkaloids (**71**–**76**). Previous bioassay of this compound library has led to the discovery of a number of protein tyrosine phosphatase-1B (PTP1B) inhibitors [19] and the cerebral protective agents [20]. In the present study, the compound library was assayed for anti-inflammatory activity by inhibiting the overproduction of the inflammatory mediator NO in LPS-activated RAW 264.7 macrophage cells, which is a classical in vitro inflammatory cell model. Furthermore, six selected bioactive compounds were further investigated for their inhibitory effect on PGE_2_ production and the modulatory mechanism on the high expression of iNOS and COX-2 proteins.

## Results and discussion

The inhibitory activity on the overproduction of NO was firstly assessed at a final concentration of 100 μM for all compounds. The level of nitrite as an indicator of NO was determined by the Griess reaction, and the cell viability was investigated by the MTT assay. Hydrocortisone sodium succinate (HSS, Tianjin Biochem Pharmaceutical Co., Ltd.), a clinically commonly used anti-inflammatory drug, was used as the positive control in this bioassay. Among the 75 tested compounds, 14 (**11**–**13**, **15**, **21**–**23**, **27**, **29**, **30**, **31**, **37**, **41**, and **42**) out of 38 β-carboline-type alkaloids showed potent NO inhibitory activity (the inhibitory rate on NO production was higher than 80%). Among them, seven compounds (**12**, **13**, **15**, **22**, **23**, **27**, and **42**) exhibited cytotoxicity (cell viability less than 50%). However, these 7 compounds did not show cytotoxicity when at a lower concentration of 50 μM. On the other hand, five compounds (**46**, **47**, **55**, **57**, and **64**) out of 31 canthinone-type alkaloids showed potent NO inhibitory activity without cytotoxicity. Among the six dimeric alkaloids, three compounds (**71**, **72**, and **76**) inhibited the NO production, but also showed cytotoxicity (Supplementary data). The results indicated the potential of a number of β-carboline-type alkaloids and canthinone-type alkaloids in the compound library as anti-inflammatory agents. Compounds (**21**, **46**, and **57**) have been reported with inhibitory NO production activities [21–23], but compounds (**11**–**13**, **15**, **22**, **23**, **27**, **29**–**31**, **37**, **41**, **42**, **47**, **55**, **64**, **71**, **72,** and **76**) were not reported as inhibitors of NO production.

The bioactive β-carboline-type alkaloids can be divided into three types, namely, type I, with an ethyl moiety at C-1 (**11**, **12**, and **13**); type II, with a double bond at C-1′ (methoxycarbonyl: **22**, **27**, **29**, **30**, and **31**; carbon–carbon double bond: **21** and **23**); and type III, with a saturated C ring and a methoxy-carbonyl at C-3 (**37**, **41**, and **42**). Compound **37** showed the strongest NO inhibitory activity, which suggest that the ethyl group at C-1 may be more contributive than other groups at the C ring. Among canthinone-type alkaloids, compounds (**46**, **47**, **57**, and **64**) without substituent groups at C-4 and C-5, were far more active than the others. In addition, comparison of the inhibition rate of 3-methyl-canthin-2,6-one (**61**) and 3-methoxy-canthin-2,6-one (**64**) indicated that the methoxy group at *N*-3 may improve the inhibitory activity.

**Fig. 1:**
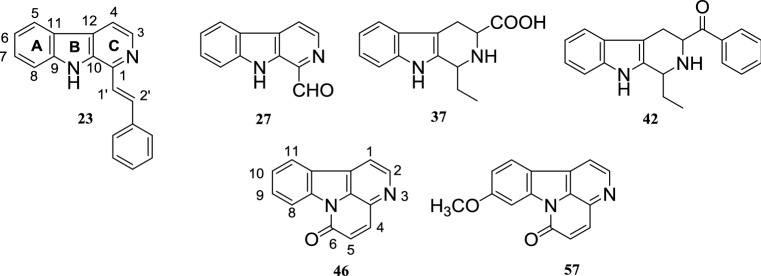
Chemical structures of selected β-carboline alkaloids (**23**, **27**, **37**, and **42**) and canthinone alkaloids (**46** and **57**)

Based on the bioassay results and the yield of compounds, six bioactive compounds, namely, benzalharman (**23**), kumujian (**27**), 1-ethyl-1,2,3,4-tetrahydro-β-carboline-3-carboxylic acid (**37**), 1-acetophenone-1,2,3,4-tetrahydro-β-carboline-3-carboxylic acid (**42**), cathin-6-one (**46**), and 9-methoxy-cathin-6-one (**57**), were selected for further investigation (Fig. 1). Compounds **27**, **46**, and **57** were isolated from *Picrasma quassioides* [12, 24], and **23**, **37**, and **42** were chemically synthetic compounds [17]. The purities of all the compounds were > 98% by HPLC–PDA and ^1^H-NMR spectroscopic analysis. Previous investigations have reported compounds **23** and **27** to be the cyclic adenosine monophosphate (AMP) phosphodiesterase inhibitors [17], and compound **46** to be an antimalarial and antitumor compound [25].

**Fig. 2:**
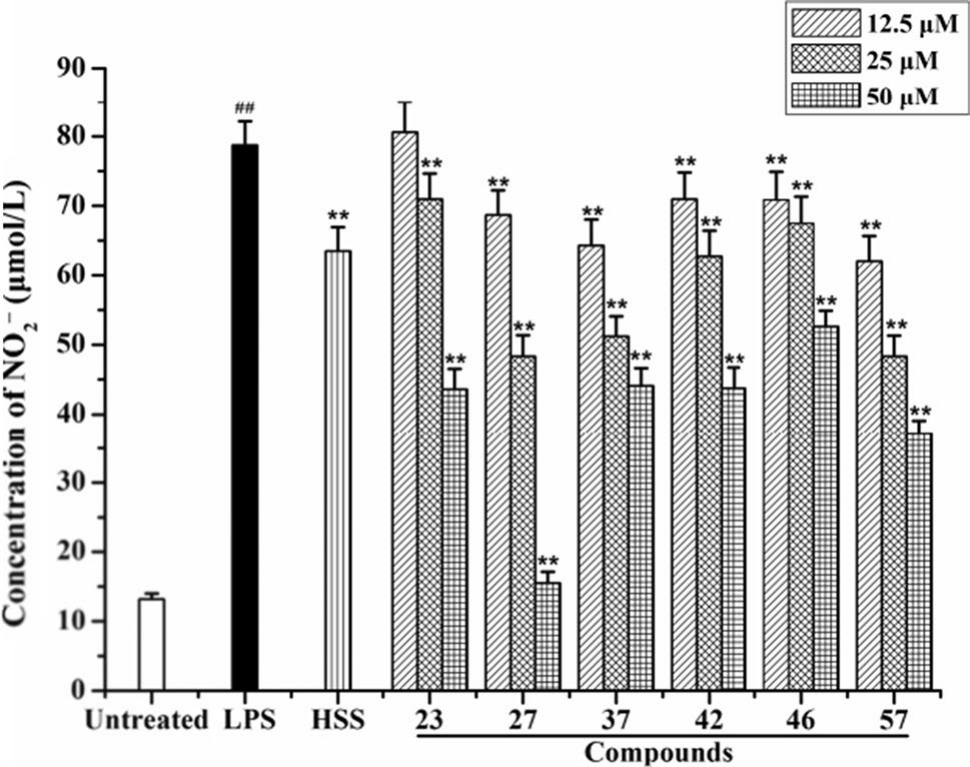
Effect of **23**, **27**, **37**, **42**, **46**, and **57** on the overproduction of NO. RAW 264.7 cells were treated by LPS (1 μg/ml) with or without test compounds (12.5, 25, and 50 μM) or HSS (50 μM) for 24 h. Cell culture supernatant (100 μl) was used to determine the level of NO. Values are expressed as mean ± SD (*n* = 3). ^##^*p* < 0.01 vs. the untreated group. ^**^p < 0.01 vs. the LPS treatment group

The positive control drug HSS showed strong inhibitory effect on the overproduction of NO induced by LPS in RAW 264.7 cells, which suggested the feasibility of using the inhibitory effect on the production of NO as an important indicator for the anti-inflammatory activity of the compound library. The six active compounds exhibited significant inhibitory activity on the release of NO in a good dose dependency; their inhibitory activity on the overproduction of NO was even stronger than that of the positive control drug at a final concentration of 50 μM (Fig. 2). At the same time, the MTT assay was used to evaluate the cytotoxicity of test compounds on the proliferation of macrophage RAW 264.7 cells. Succinate dehydrogenase converts exogenous MTT to formazan crystals which can be dissolved by dimethyl sulfoxide (DMSO). The absorbance values were measured with a microplate reader at a wavelength of 570 nm, which can indirectly reflect the viability of cells. LPS (1 μg/ml), HSS (50 μM), and six active compounds (12.5 μM, 25 μM, and 50 μM) showed no cytotoxicity on the proliferation of RAW 264.7 cells in the MTT assay (Supplementary data).

**Fig. 3:**
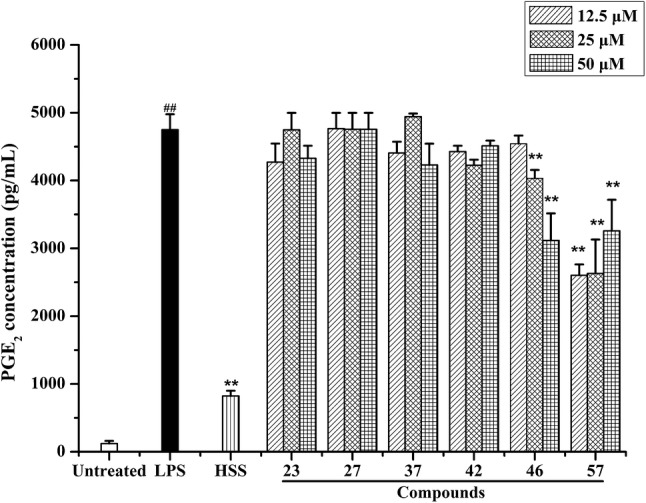
Effect of **23**, **27**, **37**, **42**, **46,** and **57** on the overproduction of PGE_2_. RAW 264.7 cells were treated by LPS (1 μg/ml) with or without test compounds (12.5, 25, and 50 μM) or HSS (50 μM) for 24 h. Cell culture supernatant (100 μl) was used to determine the level of PGE_2_. Values were expressed as mean ± SD (*n* = 3). ^##^*p* < 0.01 vs. the untreated group. ^**^p < 0.01 vs. the LPS treatment group

Furthermore, the effect of six active compounds on the release of PGE_2_ was determined by using the ELISA method. As the result, when RAW 264.7 cells were treated by LPS for 24 h, the level of PGE_2_ markedly increased comparing with the untreated group (*p* < 0.01). Four β-carboline-type alkaloids (**23**, **27**, **37**, and **42**) have no obvious inhibitory effect on the PGE_2_ secretion in LPS-activated RAW 264.7 macrophages, but two canthinone-type alkaloids (**46** and **57**) showed potent inhibitory activity against the overproduction of PGE_2_ (Fig. 3). The results clearly suggested that these two types of alkaloids show different inhibition profiles on the release of PGE_2_.

**Fig. 4:**
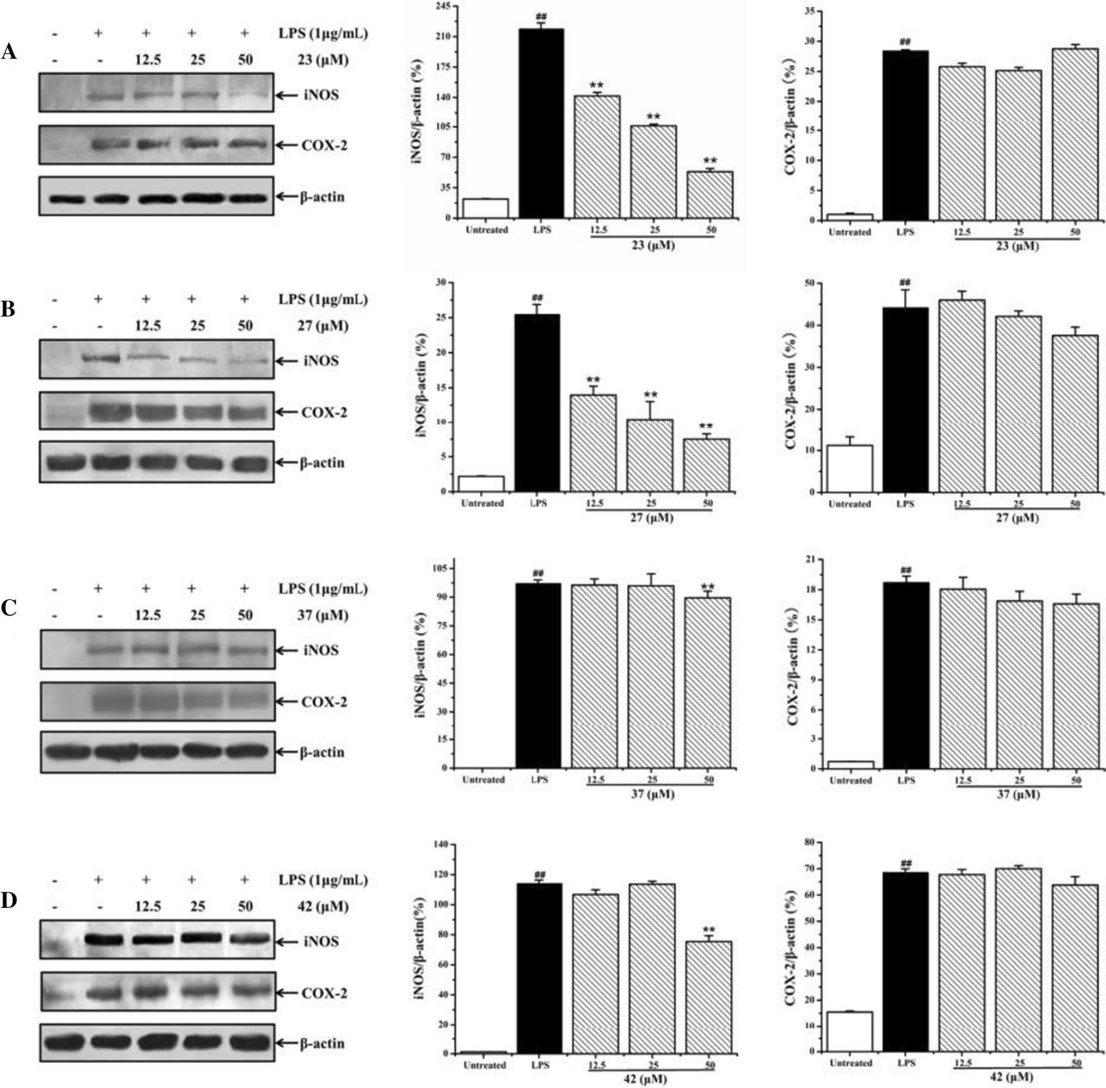
Effect of β-carboline alkaloids **23** (**a**), **27** (**b**), **37** (**c**), and **42** (**d**) on the high expression of iNOS and COX-2 proteins. RAW 264.7 cells were treated by LPS (1 μg/ml) with or without β-carboline alkaloids (12.5, 25, and 50 μM) for 24 h. The expression of iNOS and COX-2 proteins were determined by Western blot analysis. Data were normalized on the basis of β-actin levels. Values are expressed as mean ± SD (*n* = 3). ^##^*p* < 0.01 vs. the untreated group. ^**^p < 0.01 vs. the LPS treatment group

iNOS and COX-2 proteins are expressed constitutively in LPS-induced RAW 264.7 cells, and the high expression of iNOS and COX-2 promote the overproduction of NO and PGE_2_, respectively. In order to investigate the anti-inflammatory molecular mechanism of these active compounds, Western blot analysis was used to determine whether these active alkaloids can modulate the high expression of inflammatory proteins iNOS and COX-2 in LPS-activated RAW 264.7 cells. As shown in Fig. 4, when RAW 264.7 cells were treated by LPS for 24 h, iNOS and COX-2 proteins were significantly up-regulated in comparison with the untreated group (*p* < 0.01). Treatment with β-carboline-type alkaloids **23** and **27** at 12.5–50 μM prominently inhibited the high expression of iNOS in a dose-dependent manner, whereas compounds **37** and **42** weakly inhibited the high expression of iNOS protein at the high concentration of 50 μM. However, four β-carboline-type alkaloids (**23**, **27**, **37,** and **42**) showed no inhibitory effect on the expression of COX-2 protein. These results indicated that β-carboline-type alkaloids (**23**, **27**, **37**, and **42**) suppress the production of NO via down-regulation of the expression of inflammatory protein iNOS, but have no effect on the COX-2 pathway.

**Fig. 5:**
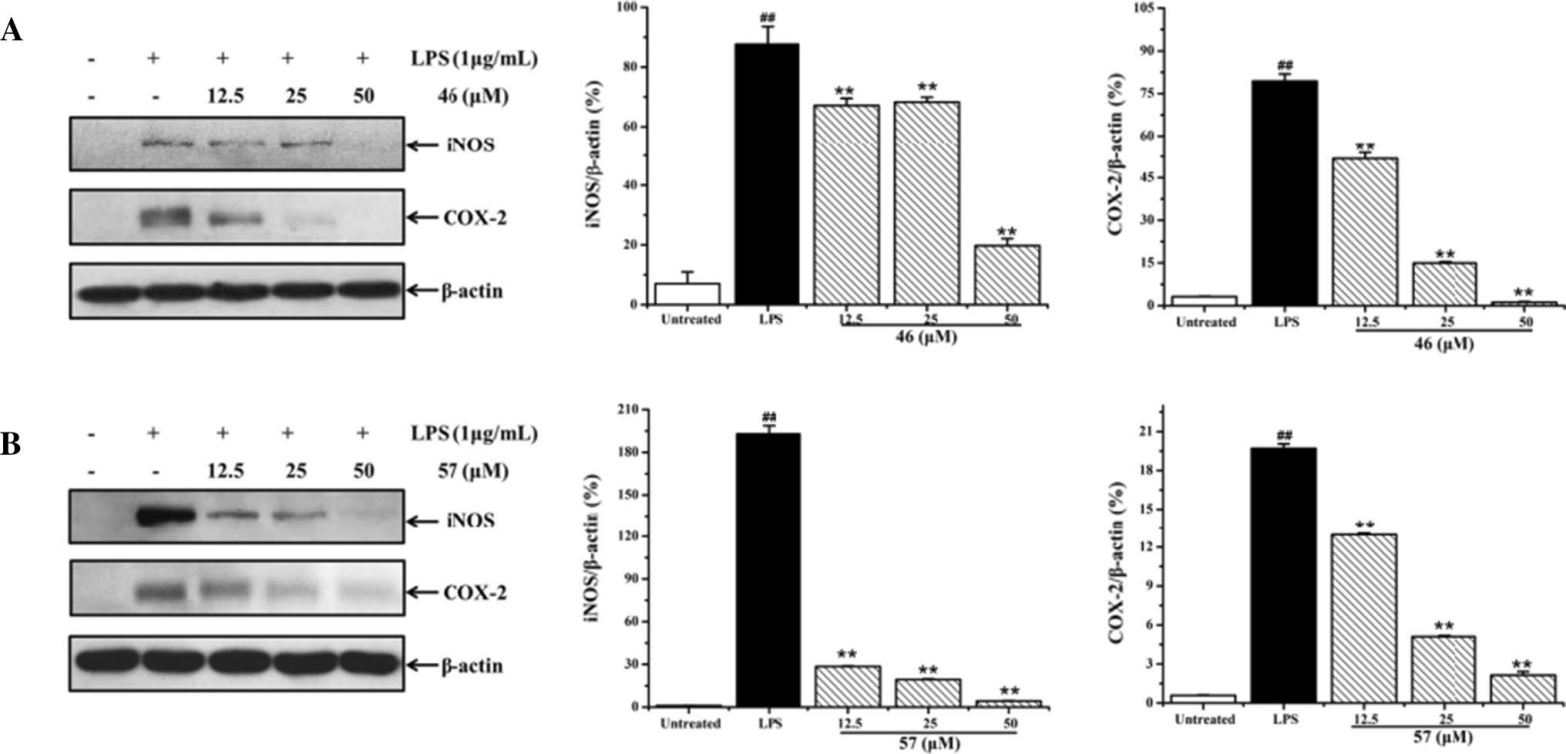
Effect of canthinone alkaloids **46** (**a**) and **57** (**b**) on the high expression of iNOS and COX-2 proteins. RAW 264.7 cells were treated by LPS (1 μg/ml) with or without canthinone alkaloids (12.5, 25, and 50 μM) for 24 h. The expressions of iNOS and COX-2 proteins were determined by Western blot analysis. Data were normalized on the basis of β-actin levels. Values were expressed as mean ± SD (*n* = 3). ^##^*p* < 0.01 vs. the untreated group. ^**^p < 0.01 vs. the LPS treatment group

In sharp contrast, in comparison with the results of β-carboline-type alkaloids, cathinone-type alkaloids **46** and **57** potently inhibited both the production of NO and the release of PGE_2_. The results of Western blot analysis indicated that cathinone-type alkaloids **46** and **57** (12.5, 25, and 50 μM) significantly down-regulated the high expression of both iNOS and COX-2 proteins (Fig. 5). All the results implied that two types of alkaloids (β-carboline-type and canthinone-type) may exert anti-inflammatory effects through different molecular mechanisms.

## Conclusion

In summary, an alkaloidal compound library from Simaroubaceae and their chemically synthesized analogues were assayed for their potential anti-inflammatory activity. The results demonstrated that a number of β-carboline-type and canthinone-type alkaloids from the compound library exhibited potent activity suppressing the overproduction of NO in LPS-activated RAW 264.7 macrophages. Based on further investigation on the selected six bioactive alkaloids, we found that two canthinone-type alkaloids suppressed the release of NO and PGE_2_ through down-regulating the expression of inflammatory proteins iNOS and COX-2. Four β-carboline-type alkaloids suppressed the production of NO via down-regulation of iNOS expression, but have no effect on the release of PGE_2_ and are independent of COX-2 pathway. Both the β-carboline-type alkaloids and the canthinone-type alkaloids have been identified as anti-inflammatory agents of Simaroubaceae plants, but these two types of natural compounds showed entirely different inhibitory profiles on the expression of iNOS and COX-2 in LPS-activated RAW 264.7 macrophages. Further detailed investigations on their modulating effect on the cellular signal transduction pathways such as NF-κB or MAPK pathways are expected to certify the differences between the mechanism of β-carboline-type alkaloids and canthinone-type alkaloids.

## Materials and methods

### General

Macrophage RAW 264.7 cells were obtained from the American Type Culture Collection (ATCC, Manassas, VA, USA). Roswell Park Memorial Institute (RPMI) 1640 medium was purchased from GE Healthcare Life Sciences. Fetal bovine serum (FBS) was purchased from NQBB International Biological Corporation. LPS (from *E. coli*), 3-(4,5-dimethylthiazol-2-yl)-2,5-diphenyltetrazolium bromide (MTT), and DMSO were purchased from Sigma–Aldrich, Inc. (St. Louis, MO, USA). HSS is a product of Tianjin Biochem Pharmaceutical Co., Ltd. (Tianjin, China). The mouse PGE_2_ ELISA kit was purchased from Shanghai Senxiong Science and Technology Industry Co., Ltd. (Shanghai, China). Mouse anti-rabbit inducible nitric oxide synthase (iNOS) polyclonal antibody (catalog no. 160862) and mouse anti-rabbit COX-2 polyclonal antibody (catalog no. 160106) were purchased from Cayman Chemical Company. Goat anti-rabbit β-actin polyclonal antibody (catalog no. sc-1616) were purchased from Santa Cruz Biotechnology, Inc. (Dallas, TX, USA).

All test compounds were dissolved in cell culture-grade DMSO at the concentration of 50 mM as a stock solution and stored at −20 °C. The stock solution was diluted to indicate concentrations by DMSO before use. The final concentration of DMSO in the medium was 0.2%. HSS, a clinical anti-inflammatory drug, was used as the positive control. HSS was dissolved in RPMI 1640 medium at the concentration of 50 mM as a stock solution and stored at − 20 °C. The stock solution was diluted to indicate concentrations by RPMI 1640 medium before use.

### Cell culture of RAW 264.7

Mouse monocyte-macrophage RAW 264.7 cells (ATCC TIB-71) were fostered in RPMI 1640 medium mixed with 10% heat-inactivated FBS at 37 °C in an incubator with 5% CO_2_. The medium was routinely replaced every day and RAW 264.7 cells were passaged until they reached about 80% of confluence.

### Nitric oxide analysis and cell viability assay

The cells were prepared at a density of 1 × 10^6^ cells/ml, and 200 μl was seeded in each well of the 96-well plates. The cells were treated by LPS (1 μg/ml; Sigma–Aldrich, St. Louis, MO, USA) with or without indicated concentrations of test compounds for 24 h. Cell culture supernatant (100 μl) was then removed to another 96 well-plate and mixed with 100 μl of Griess reagent containing equal volumes of Griess reagent A: 0.1% (w/v) of *N*-(1-naphthyl) ethylenediamine solution and Griess reagent B: 1% (w/v) sulfanilamide in 5% (v/v) H_3_PO_4_ solution (Yantai Science and Biotechnology Co., Ltd., Yantai, China). After being mixed for 10 min, the absorbance was measured at 540 nm using a microplate reader. The nitrite concentrations were calculated according to the method reported by Jin et al. [26].

After 100 μl of the cell culture supernatant was taken out for NO determination, MTT solution (5 mg/ml) was added in the original 96-well plate at the final concentration of 200 μg/ml and then incubated for 4 h. After removal of the supernatant from the 96-well plate, 150 μl of DMSO was added to dissolve the formazan. The absorbance was measured by a microplate reader at a wavelength of 570 nm, and a wavelength of 655 nm was used as reference. The untreated cells were considered to be 100% viable. Final results are expressed as percentage of viable cells in the experimental group when compared with those of the untreated group.

### Determination of PGE_2_

RAW 264.7 cells were prepared at a density of 1 × 10^6^ cells/ml, and 200 μl was seeded in each well of the 96-well plates. The cells were treated by LPS (1 μg/ml) with or without indicated concentrations of test compounds for 24 h. Cell culture supernatant (100 μl) was removed to measure the level of PGE_2_ by using a commercial mouse PGE_2_ ELISA kit (Shanghai Senxiong Science and Technology Industry Co., Ltd., Shanghai, China) according to the manufacturer’s instructions.

### Western blot analysis of iNOS, COX-2 and β-actin proteins

The cells were seeded in 60-mm cell culture dishes for 1 h, and then treated by LPS (1 μg/ml) with or without indicated compounds (12.5, 25, and 50 μM) for 24 h. The cells were washed with cold PBS and lysed in a cold lysis buffer, and the total protein was extracted from ultrasonic crushed cells. The total protein and the cellular debris were separated by centrifugation at 13,000 rpm for 6 min. The protein concentrations were determined by a commercial Bradford protein assay kit. Total protein (30 μg) was separated by 8% polyacrylamide gel electrophoresis and then transferred to nitrocellulose membranes. The membranes were blocked with Tris-buffered saline with 0.5% triton X-100 (TBS-T) containing 5% skim milk at room temperature for 4 h. The membranes were then washed with TBS-T for three times and incubated overnight at 4 °C with anti-iNOS, anti-COX-2, or anti-β-actin solution which was diluted with TBS-T. After washing with TBS-T, the membranes were incubated for 1 h at room temperature with horseradish peroxidase (HRP)-labeled goat anti-murine IgG (H+L), or HRP-labeled goat anti-rabbit IgG (H+L) as secondary antibody diluted with TBS-T, respectively. The bands were detected with an enhanced chemiluminescence system and the bands representing iNOS, COX-2, and β-actin were quantitated by DigDoc100 program (Gel pro analyzer 3.2). The levels of corresponding iNOS and COX-2 were normalized on the basis of the corresponding β-actin levels.

### Statistical analysis

The data are expressed as mean ± SD. The results were assessed by one-way analysis of variance with the SPSS 16.0 statistical program. *p* ≤ 0.05 was considered to indicate a statistically significant difference.

## Electronic supplementary material

Below is the link to the electronic supplementary material.

Supplementary data included: Chemical structures of seventy-five alkaloids, the primary screening results and cell viability

## References

[1] Chesrown SE, Monnier J, Visner G, Nick HS (1994). Regulation of inducible nitric oxide synthase mRNA levels by lipopolysaccharide, interferon-γ, TGF-β and IL-10 in murine macrophage cell lines and rat peritoneal macrophages. Biochem Biophys Res Commun.

[2] Bos CL, Richel DJ, Ritsema T, Peppelenbosch MP, Versteeg HH (2004). Prostanoids and prostanoid receptors in signal transduction. Int J Biochem Cell B.

[3] Pairet M, Engelhardt G (1996). Distinct isoforms (COX-1 and COX-2) of cyclooxygenase: possible physiological and therapeutic implications. Fund Clin Pharmacol.

[4] Lee JK, Sayers BC, Chun KS, Lao HC, Shipley-Phillips JK, Bonner JC, Langenbach R (2012). Multi-walled carbon nanotubes induce COX-2 and iNOS expression via MAP Kinase-dependent and-independent mechanisms in mouse RAW 264.7 macrophages. Part Fibre Toxi.

[5] Kim YS, Ahn CB, Je JY (2016). Anti-inflammatory action of high molecular weight *Mytilus edulis* hydrolysates fraction in LPS-induced RAW 264.7 macrophage via NF-κB and MAPK pathways. Food Chem.

[6] Liu JX, Tang JS, Zuo YH, Yu Y, Luo P, Yao XS, Dong Y, Wang PX, Liu L, Zhou H (2016). Stauntoside B inhibits macrophage activation by inhibiting NF-κB and ERK MAPK signaling. Pharmacol Res.

[7] Zhao F, Chen L, Bi CC, Zhang ML, Jiao HW, Yao XS (2013). In vitro anti-inflammatory effect of picrasmalignan A by the inhibition of iNOS and COX-2 expression in LPS-activated macrophage RAW 264.7 cells. Mol Med Rep.

[8] Schuster R, Zeindl L, Holzer W, Khumpirapang N, Okonogi S, Viernstein H, Mueller M (2017). Eulophia macrobulbon-an orchid with significant anti-inflammatory and antioxidant effect and anticancerogenic potential exerted by its root extract. Phytomedicine.

[9] Kalinski P (2012). Regulation of immune responses by prostaglandin E_2_. J Immunol.

[10] Zimmer AR, Leonardi B, Miron D, Schapoval E, Oliveira JR, Gosmann G (2012). Antioxidant and anti-inflammatory properties of *Capsicum baccatum*: from traditional use to scientific approach. J Ethnopharmacol.

[11] Fynn PM, Opoku-Boahen Y, Adukpo GE, Armah FA (2016). Anti-inflammatory and antioxidant activities of canthinone alkaloids from *Anthostema aubryanum* (Baill). Nat Prod Plant Resour.

[12] Ohmoto T, Koike K (1984). Studies on the constituents of *Picrasma quassioides* Bennet. III.. Chem Pharm Bull.

[13] Ohmoto T, Koike K (1984). Studies on the constituents of *Ailanthus altissima* Swingle. III. The alkaloidal constituents. Chem Pharm Bull.

[14] Jiao WH, Gao H, Li CY, Zhou GX, Kitanaka S, Ohmurae A, Yao XS (2010). β-Carboline alkaloids from the stems of *Picrasma quassioides*. Magn Reson Chem.

[15] Zhu CC, Deng GH, Lin CZ (2012). Chemical constituents of *Picrasma quassioides* (D. Don) Benn. Nat Prod Res Dev.

[16] Zhao W, Chen ZW, Sun JH, He Y, Liu W, Hu S, Guan ZY, Chen FL (2012). Alkaloid extraction from *Picramia quassioides* (D. Don) Benn. and safety of its compound injection. J South Agric.

[17] Sung YI, Koike K, Nikaido T, Ohmoto T, Sankawa U (1984). Inhibitors of cyclic AMP phosphodiesterase in *Picrasma quassioides* Bennet, and inhibitor activities of related β-carboline alkaloids. Chem Pharm Bull.

[18] Jiang MX, Zhou YJ (2008). Canthin-6-one alkaloids from *Picrasma quassioides* and their cytotoxic activity. J Asian Nat Prod Res.

[19] Sasaki T, Li W, Higai K, Koike K (2015). Canthinone alkaloids are novel protein tyrosine phosphatase 1B inhibitors. Bioorg Med Chem Lett.

[20] Sasaki T, Li W, Ohmoto T, Koike K (2016). Evaluation of canthinone alkaloids as cerebral protective agents. Bioorg Med Chem Lett.

[21] Zhao F, Gao Z, Jiao W, Chen L, Chen L, Yao X (2012). In vitro anti-inflammatory effects of beta-carboline alkaloids, isolated from Picrasma quassioides, through inhibition of the iNOS pathway. Planta Med.

[22] Cho SK, Jeong M, Jang DS, Choi JH (2018). Anti-inflammatory effects of canthin-6-one alkaloids from *Ailanthus altissima*. Planta Med.

[23] Ngoc PB, Pham TB, Nguyen HD, Tran TT, Chu HH, Chau VM, Lee JH, Nguyen TD (2016). A new anti-inflammatory β-carboline alkaloid from the hairy-root cultures of *Eurycoma longifolia*. Nat Prod Res.

[24] Ohmoto T, Koike K (1983). Studies on the constituents of *Picrasma quassioides* Bennet. II. On the alkaloidal constituents. Chem Pharm Bull.

[25] Dejos C, Voisin P, Bernard M, Regnacq M, Berges T (2014). Canthin-6-one displays antiproliferative activity and causes accumulation of cancer cells in the G2/M phase. J Nat Prod.

[26] Jin X, Song SQ, Wang J, Zhang QZ, Qiu F, Zhao F (2016). Tiliroside, the major component of *Agrimonia pilosa Ledeb* ethanol extract, inhibits MAPK/JNK/p38-mediated inflammation in lipopolysaccharide-activated RAW 264.7 macrophages. Exp Ther Med.

