# Correction to: Identification of β‑carboline and canthinone alkaloids as anti‑inflammatory agents but with different inhibitory profile on the expression of iNOS and COX‑2 in lipopolysaccharide‑activated RAW 264.7 macrophages

**DOI:** 10.1007/s11418-019-01282-y

**Published:** 2019-02-02

**Authors:** Pan Liu, Huixiang Li, Ruiling Luan, Guiyan Huang, Yanan Liu, Mengdi Wang, Qiuli Chao, Liying Wang, Danna Li, Huaying Fan, Daquan Chen, Linyu Li, Keiichi Matsuzaki, Wei Li, Kazuo Koike, Feng Zhao

**Affiliations:** 1 Key Laboratory of Molecular Pharmacology and Drug Evaluation (Yantai University), Ministry of Education, Collaborative Innovation Center of Advanced Drug Delivery System and Biotech Drugs in Universities of Shandong, School of Pharmacy, Yantai University, Yantai, Shandong 264005 People’s Republic of China; 2 Faculty of Pharmaceutical Sciences, Toho University, Funabashi, Chiba 274-8510 Japan; 3 Pharmacy Dispensing Center, The Affiliated Yantai Yuhuangding Hospital of Qingdao University, Yantai, Shandong 264000 People’s Republic of China; 4 School of Pharmacy, Nihon University, Funabashi, Chiba 274-8555 Japan

## Correction to: Journal of Natural Medicines (2019) 73:124–130 10.1007/s11418-018-1251-5

The article Identification of β‑carboline and canthinone alkaloids as anti‑inflammatory agents but with different inhibitory profile on the expression of iNOS and COX‑2 in lipopolysaccharide‑activated RAW 264.7 macrophages, written by Pan Liu, Huixiang Li, Ruiling Luan, Guiyan Huang, Yanan Liu, Mengdi Wang, Qiuli Chao, Liying Wang, Danna Li, Huaying Fan, Daquan Chen, Linyu Li, Keiichi Matsuzaki, Wei Li, Kazuo Koike, Feng Zhao, was originally published electronically on the publisher’s internet portal (currently SpringerLink) on 15 October 2018 without open access.

With the author(s)’ decision to opt for Open Choice the copyright of the article changed on 20 February 2019 to © The Author(s) 2019 and the article is forthwith distributed under the terms of the Creative Commons Attribution 4.0 International License (http://creativecommons.org/licenses/by/4.0/), which permits use, duplication, adaptation, distribution and reproduction in any medium or format, as long as you give appropriate credit to the original author(s) and the source, provide a link to the Creative Commons license and indicate if changes were made.

The original article was corrected.

