# Supplementary material for: Identification of β-carboline and canthinone alkaloids as anti-inflammatory agents but with different inhibitory profile on the expression of iNOS and COX-2 in lipopolysaccharide-activated RAW 264.7 macrophages
Source: J Nat Med. 2018 Oct 15;73(1):124–30. doi: 10.1007/s11418-018-1251-5 (PMC6407838; doi:10.1007/s11418-018-1251-5)
Supplement: Supplementary file 1 — Supplementary data included: Chemical structures of seventy-five alkaloids, the primary screening results and cell viability [file 11418_2018_1251_MOESM1_ESM.pdf]

Supplementary Data

Identification of β-carboline and canthinone alkaloids as anti-inflammatory agents but with different inhibitory profile on the expression of iNOS and COX-2 in lipopolysaccharide activated RAW 264.7 macrophages

Pan Liu[1,a] · Huixiang Li[1,a] · Ruiling Luan[3] · Guiyan Huang[1] · Yanan Liu[1] · Mengdi Wang[1] · Qiuli Chao[1] · Liying Wang[1] · Danna Li[1] · Huaying Fan[1] · Daquan Chen[1] · Linyu Li[1] · Keiichi Matsuzaki[4] · Wei Li[2,]* · Kazuo Koike[2] · Feng Zhao[1,]*

[1]Key Laboratory of Molecular Pharmacology and Drug Evaluation (Yantai University), Ministry of Education, Collaborative Innovation Center of Advanced Drug Delivery System and Biotech Drugs in Universities of Shandong, School of Pharmacy, Yantai University, Yantai, Shandong 264005, P. R. China

[2]Faculty of Pharmaceutical Sciences, Toho University, Funabashi, Chiba 274-8510, Japan

[3]Pharmacy Dispensing Center, The Affiliated Yantai Yuhuangding Hospital of Qingdao University, Yantai, Shandong 264000, P. R. China

[4]School of Pharmacy, Nihon University, Funabashi, Chiba 274-8555, Japan

Contents

1.0 Chemical structures of seventy-five alkaloids S3

2.0 Results S6

2.1 The inhibitory rate on NO production and the cell viability at a final concentration of 100 μM. S6

2.2 Effects of **23**, **27**, **37**, **42**, **46** and **57** on the viability of RAW 264.7 cells S9

1.0 Chemical structures of seventy-five alkaloids

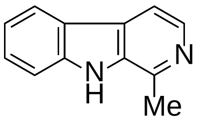
1-methyl-β-carboline (**7**)

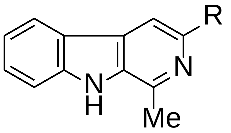
Harman-3-carboxylic acid (**8**): R = COOH
3-methoxycarbonyl-1-methyl-β-carboline (**9**): R = COOMe

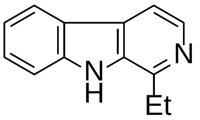
1-ethyl-β-carboline (**10**)

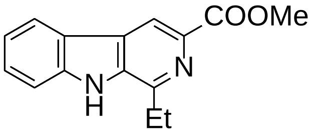
Methyl 1-ethyl-
β-carboline-3-carboxylate (**11**)

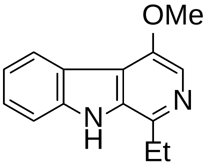
Crenatine (**12**)

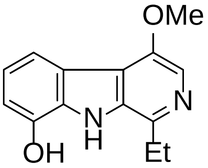
Picrasidine J (**13**)

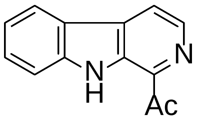
1-acetyl-β-carboline (**14**)

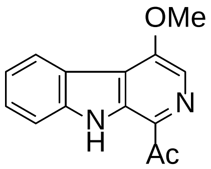
1-acethyl-4-methoxy-β-carboline (**15**)

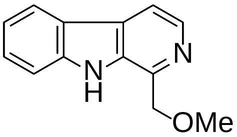
1-methoxymethyl-β-carboline (**16**)

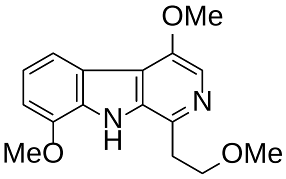
Picrasidine B (**17**)

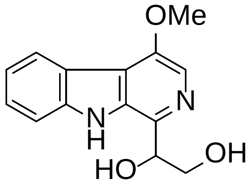
1-(1,2-ethanediol)-
4-methoxy-β-carboline (**18**)

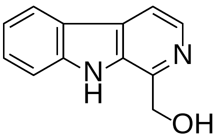
1-hydroxymethyl-β-carboline (**19**)

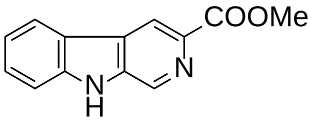
Methyl β-carboline-3-carboxylate (**20**)

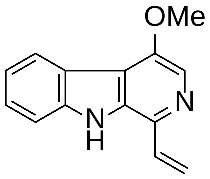
Dehydrocrenatine (**21**)

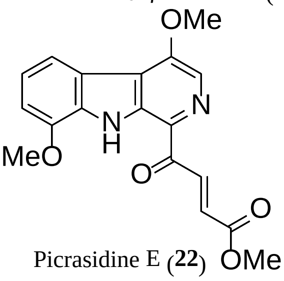
Picrasidine E (**22**)

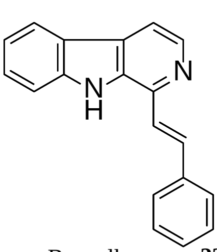
Benzalharman (**23**)

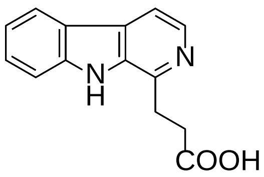
β-carboline-1-propionic acid (**24**)

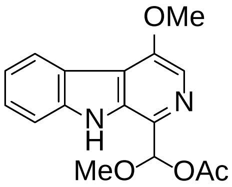
1-(2-acetoxy-1-methoxyethyl)
-4-methoxy-β-carboline (**25**)

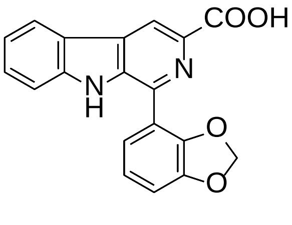
1-(1,3-benzodioxole)-
β-Carboline-3-carboxylic acid (**26**)

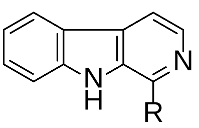
Kumujian C (**27**): R = CHO
β-carboline-1-carboxylic acid (**28**): R = COOH
Methyl β-carboline-1-carboxylate (**29**): R = COOMe
Kumujian A (**30**): R = COOEt
β-carboline-1-carboxamide (**31**): R = $CONH_2$
1-hydroxy-β-carboline (**32**): R = OH

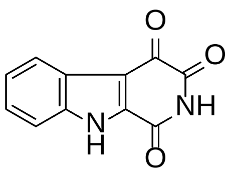
1H-Pyrido[3,4-b]indole-
1,3,4(2H,9H)-trione (**33**)

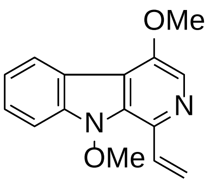
Picrasidine D (**34**)

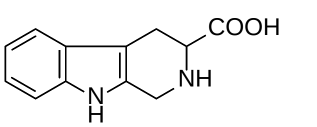

1,2,3,4-tetrahydro-β-carboline-3-carboxylic acid (**35**)

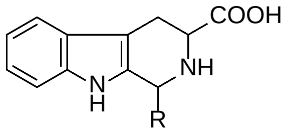

1-methyl-1,2,3,4-tetrahydro-β-carboline-3-carboxylic acid (**36**): R = Me
1-ethyl-1,2,3,4-tetrahydro-β-carboline-3-carboxylic acid (**37**): R = Et
1-propyl-1,2,3,4-tetrahydro-β-carboline-3-carboxylic acid (**38**): R = n-Pr
1-(1-methylethyl)-1,2,3,4-tetrahydro-β-carboline-3-carboxylic acid (**39**): R = i-Pr

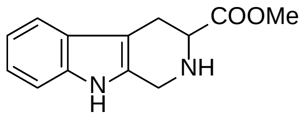

1,2,3,4-tetrahydro-β-carboline3-carboxylate (**40**)

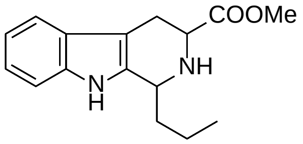

1-propyl-1,2,3,4-tetrahydro-β-carboline-3-carboxylate (**41**)

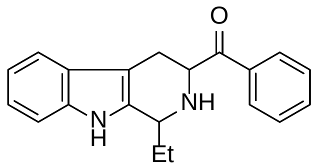

1-acetophenone-1,2,3,4-tetrahydro-β- carboline-3-carboxylic acid (**42**)

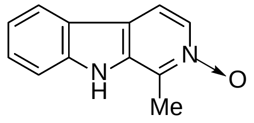

Harmanine (**43**)

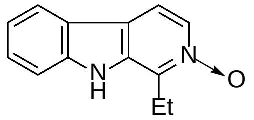

1-ethyl-harmanine N-oxide (**44**)

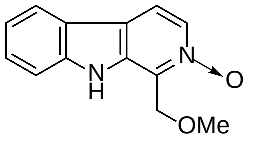

1-methoxymethyl-harmanine (**45**)

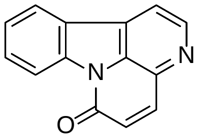

Canthin-6-one (**46**)

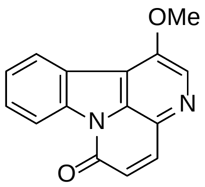

1-methoxy-canthin-6-one (**47**)

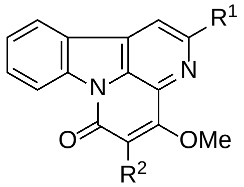

4, 5-dimethoxy-2-hydroxy-canthin-6-one (**48**): R^1 = OH, R^2 = OMe
2,5-diacetoxy-4-methoxy-canthin-6-one (**49**): R^1 = R^2 = OAc

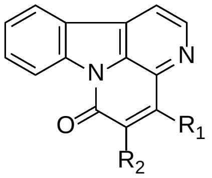

Picrasidine Q (**50**): R_1 = OH, R_2 = OMe
Nigakinone (**51**): R_1 = OMe, R_2 = OH
4, 5-dimethoxy-canthin-6-one (**52**): R_1 = R_2 = OMe
Nigakinone acetate (**53**): R_1 = OMe, R_2 = OAc

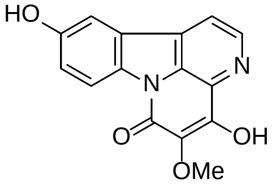

Picrasidine W (**54**)

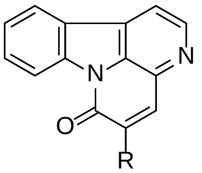

5-hydroxy-canthin-6-one (**55**): R = OH
5-methoxy-canthin-6-one (**5**): R = OMe
5-acetoxy-canthin-6-one (**6**): R = OAc

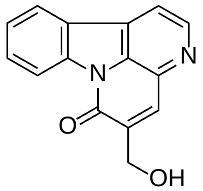

5-hydroxymethyl-canthin-6-one (**56**)

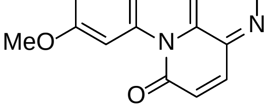

9-methoxy-canthin-6-one (**57**)

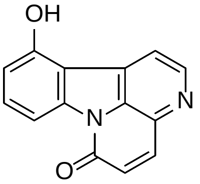

11-hydroxy-canthin-6-one (**58**)

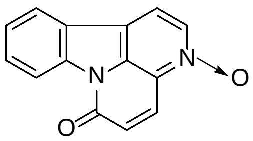

Canthin-6-one N-oxide (**59**)

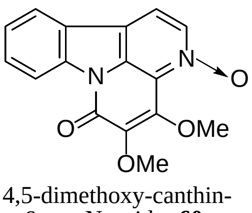

4,5-dimethoxy-canthin-6-one N-oxide (**60**)

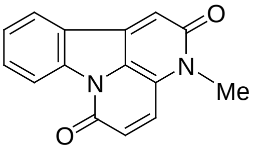

3-methyl-canthin-2,6-one (**61**)

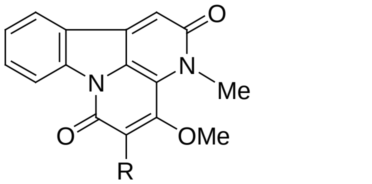

5-hydroxy-3-methyl-4-methoxy-canthin-2,6-one (**62**): R = OH
4,5-dimethoxy-3-methyl-canthin-2,6-one (**63**): R = OMe

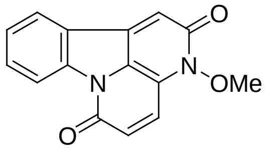

3-methoxy-canthin-2,6-one (**64**)

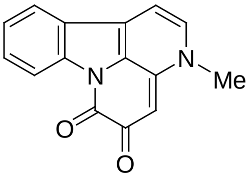

Picrasidine L (**1**)

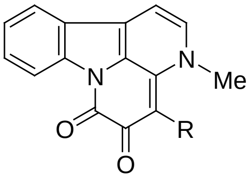

4-ethyl-3-methyl-canthin-5,6-dione (**3**): R = Et
Picrasidine O (**65**): R = OMe

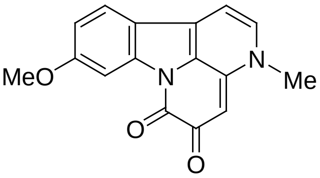

Eurycomine E (**4**)

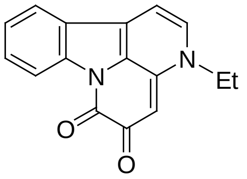

3-ethyl-canthin-5,6-dione (**66**)

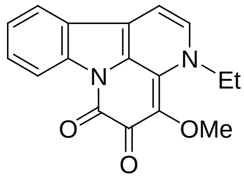

3-ethyl-4-methoxy-canthin-5,6-dione (**67**)

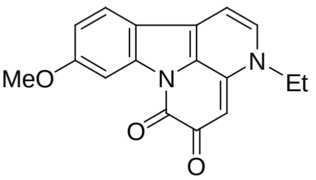

3-ethyl-9-methoxy-canthin-5,6-dione (**68**)

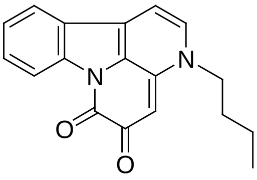

3-buthyl-canthin-5,6-dione (**69**)

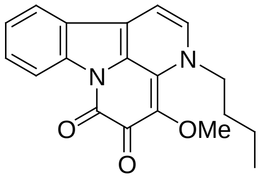

3-buthyl-4-methoxy-canthin-5,6-dione (**70**)

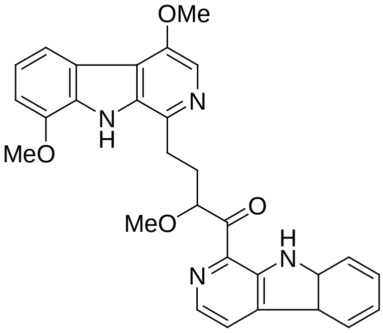

Picrasidine C (**71**)

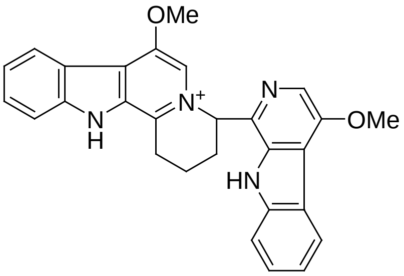

Picrasidine G (**72**)

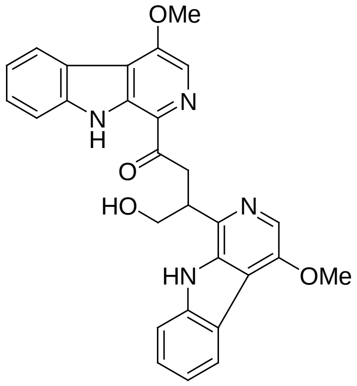

Picrasidine H (**73**)

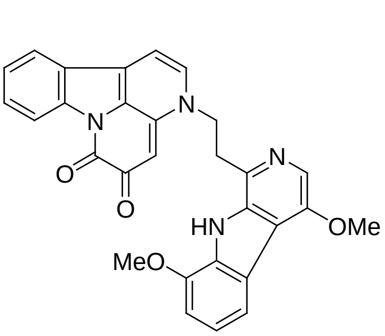

Picrasidine M (**74**)

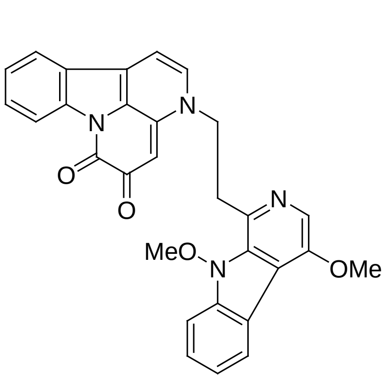

Picrasidine N (**75**)

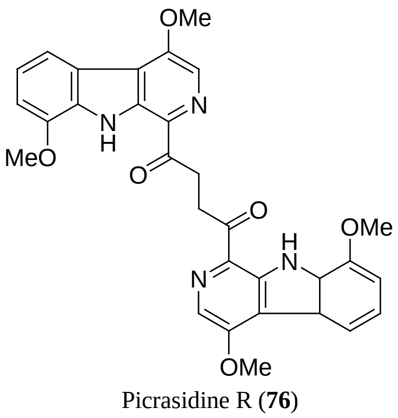

Picrasidine R (**76**)

2.0 Results

2.1 Table 1: The inhibitory rate on NO production and the cell viability at a final concentration of 100 μM.

Compounds No.	Compound name	CAS No.	Inhibition rate (%)	Cell viability (%)
**β-carboline alkaloids**				
**7**	1-methyl-β-carboline	486-84-0	37.90	102.04
**8**	Harman-3-carboxylic acid	22329-38-0	3.23	99.35
**9**	3-methoxycarbonyl-1-methyl-β-carboline	16641-82-0	12.90	103.64
**10**	1-ethyl-β-carboline	20127-61-1	29.84	75.74
**11**	Methyl 1-ethyl-β-carboline-3-carboxylate	75304-04-0	94.35	86.98
**12**	Crenatine	26585-14-8	81.45	24.85
**13**	Picrasidine J	100234-62-6	99.35	20.12
**14**	1-acethyl-β-carboline	50892-83-6	4.84	60.95
**15**	1-acethyl-4-methoxy-β-carboline	65236-63-7	93.23	59.29
**16**	1–methoxymethyl-β-carboline	55854-60-9	20.97	100.92
**17**	Picrasidine B	82652-19-5	73.39	103.20
**18**	1-(1,2-ethanediol)-4-methoxy-β-carboline	77369-99-4	76.35	92.31
**19**	1–hydroxymethyl-β-carboline	17337-22-3	37.90	100.92
**20**	Methyl β-carboline-3-carboxylate	69954-48-9	62.90	102.34
**21**	Dehydrocrenatine	26585-13-7	86.29	63.20
**22**	Picrasidine E	94530-77-5	88.71	16.21
**23**	Benzalharman	20127-62-2	93.55	13.85
**24**	β-carboline-1-propanoic acid	89915-39-9	7.26	86.27
**25**	1-(2-acetoxy-1-methoxyethyl)-4-methoxy-β-carboline	89915-41-3	35.48	85.80
**26**	1-(1,3-benzodioxole)-β-carboline-3-carboxylic acid		68.55	92.31
**27**	Kumujian C	20127-63-3	92.74	8.99
**28**	β-carboline-1-carboxylic acid	26052-96-0	77.42	103.14
**29**	Methyl β-carboline-1-carboxylate	3464-66-2	91.13	103.20
**30**	Kumujian A	72755-19-2	100.87	102.19
**31**	β-crboline-1-carboxamide	38940-60-2	84.46	69.19

**32**	1-hydroxy-β-carboline	19839-52-2	67.74	100.71
**33**	1H-Pyrido[3,4-b]indole-1,3,4(2H,9H)-trione	16641-79-5	27.42	75.86
**34**	Picrasidine D	88142-62-5	1.55	102.63
**35**	1,2,3,4-tetrahydro-β-carboline-3-carboxylic acid	6052-68-2	11.29	84.02
**36**	1-methyl-1,2,3,4-tetrahydro-β-carboline-3-carboxylic acid	5470-37-1	4.35	91.60
**37**	1-ethyl-1,2,3,4-tetrahydro-β-carboline-3-carboxylic acid		100.87	77.28
**38**	1-propyl-1,2,3,4-tetrahydro-β-carboline-3-carboxylic acid		1.61	102.49
**39**	1-(1-methylethyl)-1,2,3,4-tetrahydro-β-carboline-3-carboxylic acid		19.35	102.40
**40**	1,2,3,4-tetrahydro-β-carboline-3-carboxylate		24.19	83.31
**41**	1-propyl-1,2,3,4-tetrahydro-β-carboline-3-carboxylate		85.48	100.74
**42**	1-acetophenone-1,2,3,4-tetrahydro-β-carboline-3-carboxylic acid		102.42	9.59
**43**	Harmanine	24223-07-2	62.90	98.11
**44**	1-ethyl-harmanine	90686-26-3	4.84	72.78
**45**	1-methoxymethyl-harmanine	119995-26-5	57.26	91.60
**Canthinone alkaloids**				
**46**	Canthin-6-one	479-43-6	95.97	97.51
**47**	1-methoxy-canthin-6-one	60755-86-4	83.06	99.88
**48**	4, 5-dimethoxy-2-hydroxy-canthin-6-one		5.00	72.43
**49**	2,5-diacetoxy-4-methoxy-canthin-6-one		70.16	56.69
**50**	Picrasidine Q	101219-61-8	58.87	100.89
**51**	Nigakinone	18110-86-6	37.10	100.36
**52**	4,5-dimethoxy-canthin-6-one	18110-87-7	1.45	92.19
**53**	Nigakinone acetate	18211-86-4	0.77	79.17
**54**	Picrasidine W	155416-28-7	8.06	101.51
**55**	5-hydroxy-canthin-6-one	64118-73-6	95.27	88.80
**5**	5-methoxy-canthin-6-one	15071-56-4	57.26	102.57
**6**	5-acetoxy-canthin-6-one	99964-80-4	20.97	103.76

**56**	5-hydroxymethyl-canthin-6-one	89915-37-7	62.90	77.04
**57**	9-methoxy-canthin-6-one	74991-91-6	92.74	50.30
**58**	11-hydroxy-canthin-6-one	75969-83-4	1.26	78.82
**59**	Canthin-6-one N-oxide	60755-87-5	52.42	103.99
**60**	4,5-dimethoxy-canthin-6-one N-oxide		12.10	100.00
**61**	3-methyl-canthin-2,6-dione	82652-21-9	0.81	88.64
**62**	5-hydroxy-3-methyl-4-methoxy-canthin-2,6-dione	129724-30-7	0.97	100.06
**63**	4,5-dimethoxy-3-methyl-canthin-2,6-dione		0.26	94.20
**64**	3-methoxy-canthin-2,6-dione	74991-92-7	80.65	100.12
**1**	Picrasidine L	96405-70-8	3.52	96.57
**3**	4-ethyl-3-methyl-canthin-5,6-dione		7.26	103.79
**65**	Picrasidine O	101219-63-0	7.26	82.72
**4**	Eurycomine E	119935-09-0	3.06	89.82
**66**	3-ethyl-canthin-5,6-dione		-4.03	92.31
**67**	3-ethyl-4-methoxy-canthin-5,6-dione	221149-66-2	1.71	99.23
**68**	3-ethyl-9-methoxy-canthin-5,6-dione	96405-77-5	2.42	90.89
**69**	3-buthyl-canthin-5,6-dione	96405-73-1	3.71	92.07
**70**	3-buthyl-4-methoxy-canthin-5,6-dione	221149-70-8	8.87	99.88
**Dimeric alkaloids**				
**71**	Picrasidine C	88142-61-4	73.39	8.64
**72**	Picrasidine G	112503-90-9	85.48	9.59
**73**	Picrasidine H	119935-11-4	24.19	93.49
**74**	Picrasidine M	99964-79-1	45.97	67.46
**75**	Picrasidine N	101219-62-9	52.42	70.77
**76**	Picrasidine R	106154-68-1	102.36	75.62
	Hydrocortisone sodium succinate		75.07	97.98

Note: The seventy-five compounds were screened for their inhibitory activities against NO overproduction at a final concentration of 100 μM. The cell viability was assayed by MTT method to evaluate the cytotoxicity.

2.2 Figure 1. Effects of **23**, **27**, **37**, **42**, **46** and **57** on the viability of RAW 264.7 cells

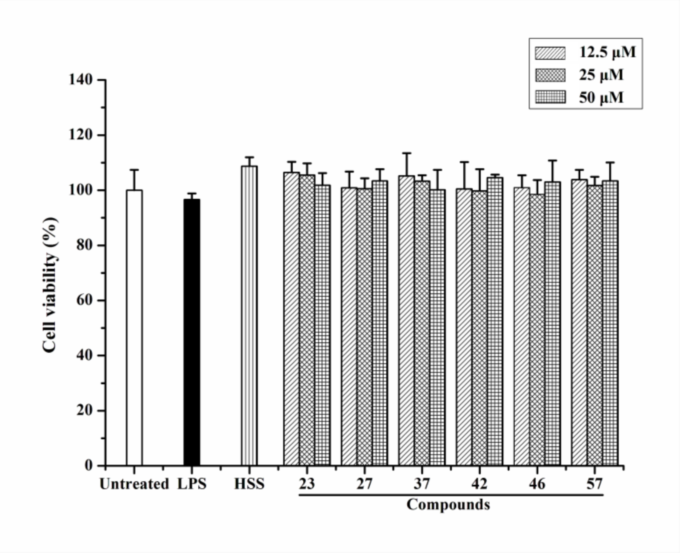

RAW 264.7 cells were treated by LPS (1 μg/mL) with or without test compounds (12.5, 25, 50 μM) or hydrocortisone sodium succinate (HSS, 50 μM) for 24 h. An MTT solution (final concentration is 200 μg/mL) was added, and the cells were incubated for another 4 h at 37°C. After removing the supernatant, 100 μL of DMSO were added to dissolve the formazan. The absorbance of each group was measured by using a microplate reader at a wavelength of 570 nm. The control group, consisting of untreated cells, was considered as having 100% of viable cells. Results are expressed as percentage of viable cells when compared with the control group. The data are expressed as mean ± SD (n=3).
